# The Multifaceted Roles of MYC2 in Plants: Toward Transcriptional Reprogramming and Stress Tolerance by Jasmonate Signaling

**DOI:** 10.3389/fpls.2022.868874

**Published:** 2022-04-25

**Authors:** Cheng Song, Yunpeng Cao, Jun Dai, Guohui Li, Muhammad Aamir Manzoor, Cunwu Chen, Hui Deng

**Affiliations:** ^1^College of Biological and Pharmaceutical Engineering, West Anhui University, Lu’an, China; ^2^Wuhan Botanical Garden, Chinese Academy of Sciences, Wuhan, China; ^3^College of Life Science, Anhui Agricultural University, Hefei, China

**Keywords:** JA signaling, stress tolerance, transcriptional regulation, crosstalk, secondary metabolism

## Abstract

Environmental stress is one of the major restrictions on plant development and foodstuff production. The adaptive response in plants largely occurs through an intricate signaling system, which is crucial for regulating the stress-responsive genes. Myelocytomatosis (MYC) transcription factors are the fundamental regulators of the jasmonate (JA) signaling branch that participates in plant development and multiple stresses. By binding to the *cis*-acting elements of a large number of stress-responsive genes, JA-responsive transcription factors activate the stress-resistant defense genes. The mechanism of stress responses concerns myriad regulatory processes at the physiological and molecular levels. Discovering stress-related regulatory factors is of great value in disclosing the response mechanisms of plants to biotic or abiotic stress, which could guide the genetic improvement of plant resistance. This review summarizes recent researches in various aspects of MYC2-mediated JA signaling and emphasizes MYC2 involvement in plant growth and stress response.

## Introduction

Phytohormones are a group of signaling molecules that are essential to perceive changes in the external environment, regulate their growth status, defend themselves against insects and maintain survival (Santner and Estelle, 2009; Dubois et al., 2018). JA is often used as a plant growth regulator as well as a signaling molecule for plant defense responses (Mao et al., 2017). Biotic and abiotic stresses have always been the vital factors in restricting the yield of food products. The adaptive responses are mainly achieved by simulating the transcription of a large number of downstream genes through complex signaling pathways (Sabagh et al., 2022). JA mediated plant development and a variety of biotic stresses caused by physical injury, pathogen attack, and insect feeding, as well as several abiotic challenges such as low temperature, drought, excessive salinity, and UV radiation (Verma et al., 2016; Yang et al., 2019). To protect against external invasion, plants perceive and transmit JA cascade signals through a variety of regulatory factors that are over-defensive and may hamper plant growth and development (Zander et al., 2020). MYC transcription factors could bind to the promoter regions of several targeted-genes, which affects their expression that help plants deal with stresses. In Arabidopsis, MYC2-mediated activation of JA signaling is precisely regulated by its repressor, the JASMONATE-ZIM DOMAIN (JAZ) protein, and a subunit of the Mediator complex (MED25) protein (Wu et al., 2020). Jasmonic acid-isoleucine conjugate (JA-Ile), as an active molecule, binds to the subunit of the Skp1/Cullin/F-box (SCF^COI1^) ubiquitin ligase and transmits downstream signaling (Wasternack and Xie, 2010). In the absence of external stress, JAZ protein binds to MYC2 and other transcription factors, rendering them inactive and devoid of triggering the transcription of downstream genes. MYC2 is repressed by JAZ, resulting in the closure of the downstream regulatory switch. Once JA-Ile is present as an activating signal, CORONATINE-INSENSITIVE 1 (COI1), JA-Ile and JAZ proteins form a co-receptor complex. COI1 regulates ubiquitin-dependent JAZ degradation via the 26S proteasome, releasing MYC2 from repression (Thines et al., 2007). Some studies demonstrated that MYC2-mediated JA signaling had an influence on the transcription reprogramming of a variety of stress-responsive genes (Zander et al., 2020). Given the critical role of MYC transcription factors, this review highlights the regulatory functions of MYCs at multiple levels and dwells on the activation and reduction mechanisms of JA signaling.

## Components and Structural Functions of MYC2 Transcription Factors

MYC proteins are the core transcription factors in JA signaling pathway. As a branch of the basic helix-loop-helix (bHLH) superfamily, the MYC family is widely present in bryophytes and angiospermae (Peñuelas et al., 2019). The MYC-related TFs between *Arabidopsis thaliana* and the liverwort *Marchantia polymorpha* had an early-diverging lineage. The evolutionary relationship suggested MYC function originally occurred in charophycean algae and hence predates the evolutionary appearance of any other jasmonate pathway component. Plants have a large number of bHLH family members that mainly involved in plant growth, stress response, and secondary metabolic processes (Heim et al., 2003; Toledo-Ortiz et al., 2003). Their bHLH genes are more closely related to the group B in the animal members. *AtbHLHs* were categorized into 12 families and then further classified based on conserved domains, with 14 new members found (Toledo-Ortiz et al., 2003). Currently, 158 and 173 bHLH transcription factors have been identified in *A. thaliana* and *O. sativa*, respectively (Pires and Dolan, 2010).

MYC-like transcription factors have three subfamilies, namely IIId, IIIe, and IIIf bHLHs. MYC2 belongs to the IIIe bHLH clade. In *A. thaliana*, IIIe bHLH members consist of *AtMYC2*, *AtMYC3*, *AtMYC4*, and *AtMYC5*. *AtMYC2* was discovered from the screening of *jasmonate-insensitive* (*jin*) mutants. Earlier study indicated that *jin1* and *jin4* mutant showed insensitivity to exogenous JA stimulation (Berger et al., 1996). AtMYC2 positively regulated both JA-mediated wounding response and negatively regulates JA-mediated resistance to saprophytic pathogens (Lorenzo et al., 2004). In addition to its high affinity for the canonical G-box, MYC2 binds to the G-box mutants 5′-CACATG-3′ and 5′-CACGTT-3′ with low affinity (Dombrecht et al., 2007). The base types at both ends of the G-box affect MYC2 binding, and the number of thymine residing at the C-terminus of the G-box could affect the binding ability of MYC2 (Figueroa and Browse, 2012).

**FIGURE 1:**
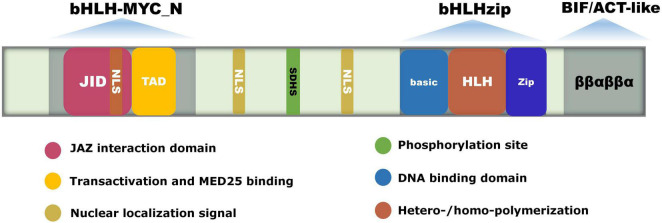
The gene structure and conserved domains of MYC2. bHLH-MYC_N, bHLH-zip, and BIF/ACT-like are the three conserved domains of MYC2. The bHLH domain at the C-terminus is composed of the Basic and HLH domains, and the N-terminus contains the JID and TAD domains. The Basic domain is responsible for binding to the G-box in the promoter of the targeted gene. The HLH domain is responsible for forming homo- or heterodimers. The JID domain binds to the Jas domain of the JAZ protein, and the TAD domain is responsible for transactivation and interactions with the MED25 subunit. The BIF structural domain contains a ββαβββα structure similar to the ACT domain, and is responsible for transactivation and homodimer formation. NSL is the nuclear localization signaling structure. The conserved amino acid SDHS is the phosphorylation site.

MYC2 included a 60-amino acid conserved bHLH domain at the C-terminus, which was composed of a Basic and an HLH domain, along with a JID domain and a TAD domain at the N-terminus (Goossens et al., 2015). The Basic domain consists of 15–20 basic amino acids and is mainly responsible for binding the G-box. The HLH domain consists of a loop region connecting two α-helices composed of hydrophobic amino acids, which are responsible for forming homo- or heterodimers. The JID domain is responsible for interacting with JAZ proteins, while the TAD domain is responsible for transcriptional activation and interactions with MED25 (Figure 1). MYC2 functions by binding to DNA through its basic domain (Lian et al., 2017). The crystal structure of the bHLH domain of MYC2 protein combined with DNA shows that two homodimers form parallel shoulder-to-shoulder tetramers reversely arranged in the MYC2-DNA structure, while MYC3 can only form homodimers (Lian et al., 2017). In contrast to MYC3, MYC2 has a stronger activation capacity. This is because the tetramerized MYC2 can mediate the formation of DNA looping structures, which in turn increases the ability of MYC2 to bind DNA and activate gene expression (Kazan and Manners, 2013).

## Regulatory Mechanisms of MYC2 Transcription Factors in the Jasmonate Signaling Pathway

Although JA is the initiator that activates the JA signaling pathway, the real functional component is JA-Ile (Suza and Staswick, 2008). JA-Ile is synthesized by an ATP-dependent jasmonate-amino acid amidase encoded by *JASMONATE RESISTANT 1 (JAR1)* and whose product, (3R,7S)-jasmonoyl-L-isoleucine, is the most biologically active JA component. JAZ proteins, as a type of repressors, could interact with other transcription factors or co-repressors to block downstream signaling. The TIFY family included three subfamilies JAZ, Zinc-finger protein expressed in Inflorescence Meristem (ZIM) and PEAPOD (PPD), among which JAZ did not contain DNA binding domains and required reciprocal interactions to perform its functions (Bai et al., 2011). There were three conserved domains within JAZ, including NT, ZIM, and Jas (Zhang et al., 2017). The NT domain at the N-terminus interacted with the GIBBERELLIC ACID INSENSITIVE (DELLA) protein (Wager and Browse, 2012). The ZIM domain contained the TIFY conserved motif TIF[F/Y]XG, which was responsible for homo- or heterodimer formation of the JAZ protein and interacts with the co-repressor Novel Interactor of JAZ (NINJA) (Pauwels et al., 2010). The C-terminal Jas domain could directly interact with the JID domain of MYC2 or COI1, which was responsible for the degradation of JAZ. Some JAZ members also contained an EAR motif at the N-terminus, which interacted directly with its co-repressor TOPLESS (TPL). Those proteins that did not have an EAR motif could not directly interact with TPL and required the participation of the NINJA protein. Due to the EAR motif in NINJA, JAZ, and TPL formed a JAZ-NINJA-TPL complex to exert its transcriptional repression. TPL protein is a member of the Gro/Tup1 family. Sixteen Groucho/Thymidine uptake 1 (Gro/Tup1) members from Arabidopsis are divided into two major groups. As co-repressors of MYC-regulon, TOPLESS/TOPLESS-Related/WUSCHELINTERACTING PROTEIN (TPL/TPR/WSIP) and LEUNIG/LEUNIG_HOMOLOG (LUG/LUH) participated in organ development, stem cell maintenance, environmental physiology, and so on (Ma et al., 2017). Although JAZ probably suppressed MYC function by interacting with MYC, some studies demonstrated that JAZ–TF transcriptional modules could control discrete branches of the JA-responsive genes in an MYC-independent manner (Cheng et al., 2011; Major et al., 2017). JASMONATE-ASSOCIATED MYC2-LIKE (JAM) and MYC2-TARGETED BHLH (MTB) from IIId bHLHs inhibited JA response by binding competitively to the promoters of MYC2-targeted genes (Sasaki-Sekimoto et al., 2013; Song et al., 2013; Wasternack, 2019).

**FIGURE 2:**
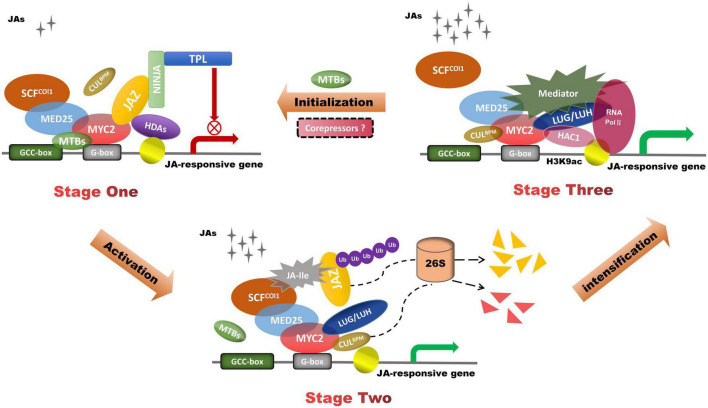
JAZ and MED25 mutually regulate JA-induced transactivation of MYC2-targeted genes. In Stage one, the MED25–MYC2 interaction is relatively weak because JAZ competes with MED25 for interaction with MYC transcription factors. In Stage two, COI1–JAZ co-receptor complex leads to proteasome-dependent degradation of JAZ repressors, which participate in the degradation of JAZ by 26S proteasome. In Stage three, MED25 recruits Pol II and LUH to the promoter of the MYC2-targeted gene, thereby regulating their expression. LUH enhanced the interactions of MYC2 coupled with its co-activators MED25 and HAC1. MTBs competitively interact with MYC2-targeted genes and impair the MED25–MYC2 complex. CUL3^BPM^ reduce MYC levels by the ubiquitination of MYC TFs.

JA-mediated MYC2 activation is fine-tuned by JAZ and the mediator complex MED25 (Figure 2). In addition to interacting with MYC2, MED25 also interacts with the receptor COI1 and a subset of JAZ repressors like NINJA and TPL (Zhai and Li, 2019; Zhai et al., 2020). In the repression phase, JAZ and its co-repressors compete with MED25 for the interaction with MYC2. In the activation phase, JA-Ile acts as molecular docking to facilitate the COI1–JAZ conjugation, which leads to proteasome-dependent degradation of JAZ repressors (Zhang F. et al., 2015). In the response phase, MED25 interacts with MYC2 and recruits HISTONE ACETYLTRANSFERASE OF THE CBP FAMILY1 (HAC1) as well as RNA polymerase II (Pol II) to the promoters of MYC2-targeted genes, thereby activating their expression (An et al., 2017). HAC1 regulates the transcription of JA-responsive genes by epigenetically controlling the H3K9 acetylation of MYC2-target genes. The Gro/Tup1 family members LUG and LUH activate MYC2-mediated transcription of *JAZ2* and *LOX2* through the mediator complex and HAC1 (You et al., 2019). LUH promotes JA-dependent enhancement of protein interactions between MYC2 and its coactivators MED25 and HAC1. MED25 recruits LUH to the promoter of the MYC2-targeted gene. MED25 and MYC2 can activate the expression of MYC2-TARGETED BHLH (MTB), which reduces the expression of JA-responsive genes. MTBs negatively regulate JA-responsive transcriptional reprogramming by weakening the stability of MYC2-MED25 complex (Liu et al., 2019). Accompanied by the deactivation of the JA signal, the status returns to the repression phase. MYC2-mediated transcription of downstream targets is not only dependent on the activation of MED25, but also associated with enhancers that assist the transcription of promoters, such as JA enhancers (JAEs) (Wang H. et al., 2019). JA mediates MED25 to regulate the chromatin looping formation between JAE and its promoter, while MYC2 uses the JAE element to regulate its own expression. The JAE (ME2 element) in the MYC2 locus positively regulates MYC2 expression in the short-term JA response and negatively regulates MYC2 in the persistent JA response. Recent studies had emerged CUL3-based E3 ubiquitin ligases(CUL3^BPM^) roles in the ubiquitination of MYC proteins (Chico et al., 2020). JA stabilized BPM3 to construct a negative feedback regulatory loop to mediate the expression of MYC.

Depending on the JID domain, AtMYC2 can interact with most JAZs, and SlMYC2 can interact with all JAZs in tomato (Fernández-Calvo et al., 2011; Du et al., 2017). MYC3 interacted with JAZ9 not only in the JID domain but also in the TAD domain. This suggests that MYC2 utilizes the same and overlapping regions to interact with JAZ and MED25. The result that JAZ9 competitively interacted with MED25 and MYC2 explained why JAZ and MED25 had both antagonistic and synergistic effects on MYC2 transcription (Zhang F. et al., 2015). Changes in conserved amino acids would bring MYC2 and MYC3 out of repression and increased their transcriptional activities (Goossens et al., 2015). The MYC2^*D*105*N*^ mutant was constructed by introducing aspartic acid at position 105 of the MYC2 amino acid, which merely interacted with JAZ1 and JAZ10 with significantly reduced binding capacity. Compared with MYC2, MYC3, and MYC4 have overlapping roles and their uniqueness in response to JA signaling and downstream regulation. MYC3 and MYC4 could form heterodimers and tetramers with MYC2, respectively. The functional deletion of MYC3 and MYC4 partially attenuated the capacities of JA response, while increased the insensitivity in *myc2* mutants (Fernández-Calvo et al., 2011). In contrast to MYC3, MYC2 had a faster and higher levels of inducible response to JA (Lian et al., 2017). Taken together, MYC3 and MYC4 may play a part in the activation of JA signaling, which requires collaboration with MYC2 to regulate JA-mediated stress responses at different levels.

## MYC2 is Involved in the Regulation of Plant Growth and Development

**FIGURE 3:**
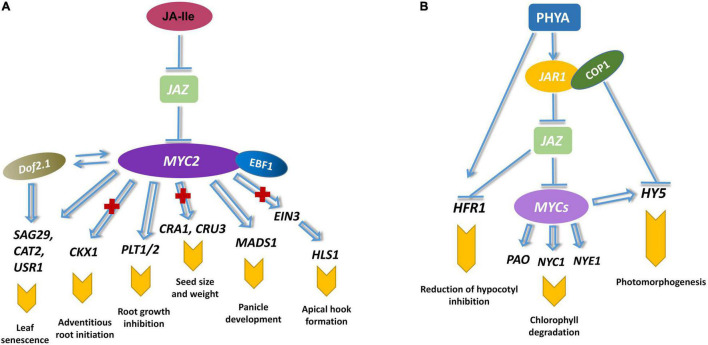
MYC2 regulates plant growth and photomorphogenesis. **(A)** MYC2 mediates JA-regulated root growth and meristem development. MYC2 inhibits adventitious root initiation by reducing the expression of *CKX1* (Dob et al., 2021). JA-induced division and stem cell differentiation in meristematic tissue is mediated by MYC2, which interacts with PLT to suppress the expression of PLT-targeted genes (Chen et al., 2011). MYC2 restrains seed development by regulating the expression of seed storage protein CRA1 and CRU3 (Gao et al., 2016). MYC2 and EIN3 additionally controlled apical hook development by interacting with EBF1 and repressing the expression of *HLS1* (Zhang et al., 2014). Senescence-associated genes are mutually enhanced by MYC2 and Dof proteins (Zhuo et al., 2020). MYC2 accelerates leaf senescence by raising the transcription of *SAG29*, *CAT2*, and *USR1* (Qi et al., 2015b; Zhang Z. et al., 2020). **(B)** MYCs are involved in the photomorphogenesis of plants. PHYA regulates the downstream of JA signaling and ultimately affects the activation of MYCs (Chico et al., 2014). PHYA control the hypocotyl inhibition and chlorophyll reduction through JA-mediated HFR1 and HY5 (Chakraborty et al., 2019). MYCs regulate JA-induced Chl degradation by directly activating some Chl catabolic genes (*PAO, NYC1, NYE1*) (Zhu et al., 2015).

MYC2 not only regulates JA-mediated growth, development and resistance, but also participates in light signaling, circadian rhythm and cross-talk with other hormones (Figure 3). MYC2 restrained leaf vein development by reducing auxin biosynthesis (Dombrecht et al., 2007; Huang et al., 2017). MYC2 negatively regulates the development of leaf veins. JA also mediates MYC2 to inhibit the cell division of the primary root meristem (Chen et al., 2011). MYC2 binds to the G-box promoter of *PLETHORA (PLT)* in response to JA signaling, resulting in the reduction of MYC2-targeted genes. JA-mediated root growth is suppressed by *MYC2*. MYC2 is also involved in seed size, seed weight, and the formation of seed storage proteins. AtMYC2 has been shown to be positively correlated with seed storage rate at various stages (Gao et al., 2016). A recent study has demonstrated that MYC2 could inhibit adventitious root initiation by suppressing *CYTOKININ OXIDASE/DEHYDROGENASE1* (*CKX1*) and enhancing the transcription of *APETALA2.6 LIKE* (*RAP2.6L*) (Dob et al., 2021). MYC2 and ETHYLENE INSENSITIVE3 (EIN3) synergistically controlled plant development and JA response (Zheng et al., 2017). MYC2 physically interacted with EIN3 to reduce *HOOKLESS1* (*HLS1*) expression or binding to the promoter of *EIN3 BINDING F-BOX PROTEIN1* (*EBF1*) to induce its expression (Zhang et al., 2014).

Light is indispensable for plant development and morphogenesis. MYC2 participated in photomorphogenesis by activating *phytochrome A* (*phyA*), which led to JA biosynthesis that recruited COI1 from the degradation of JAZ (Hsieh and Okamoto, 2014). Degradation of JAZ repressors results in the release of MYC transcription factors. At low R/FR ratios, *phyA* and *myc2* mutants both showed decreased hypocotyl inhibition, which suggested MYC2 was responsible for far-red light response. Additionally, *myc2* and *phyA* mutants exhibited a significant hypocotyl elongation of sheltering effect (Chico et al., 2014). HYPOCOTYL 5 (HY5) is a basic leucine-zipper (bZIP) transcription factor involved in photomorphogenesis at different wavelengths. *hy5* mutants exhibit reduced hypocotyl elongation and chlorophyll accumulation. MYC2 and HY5 regulate seedling development under blue light irradiation (Chakraborty et al., 2019). MYC2 negatively regulates the expression of *HY5* by binding to the promoter E-box. HY5 acts toward *MYC2* in different patterns, implying that they coordinate seedling growth in a mutually antagonistic way. Chlorophyll (Chl) degradation is a characteristic event during green organ senescence or maturation. JA-induced Chl degradation could be mediated by directly activating some Chl catabolic genes, such as *pheophorbide a oxygenase* (*PAO*), *NON-YELLOW COLORING 1* (*NYC1*), and *NON-YELLOWING 1* (*NYE1*) (Zhu et al., 2015). The *myc* and *phyB* mutants exhibit similar phenotypes in delaying seed development and hypocotyl growth. Overexpression of *MYC2* in *phyB* mutants partially reduces the prolonged hypocotyl phenotype (Ortigosa et al., 2020). By binding to the promoter of *HY5*, MYC2 and MYC3 activate the expression of the red light-responsive gene *HY5*. In addition to light signaling, the expression of *MYC2* is regulated by circadian rhythm. MYC2 interacts with TIME FOR COFFEE (TIC) resulting in its degradation by the ubiquitination of 26S proteasome. The *tic* and *myc2* mutants exhibited opposite JA-responsive phenotypes (Shin et al., 2012).

Floral initiation is a sophisticated and complex process, and the timing of flowering is synergistically regulated by a variety of factors such as temperature, light, and hormones. MYC2 is involved in JA-mediated inhibition of flowering (Wang et al., 2017). The *myc2/3* and *myc2/4* double mutants exhibited an earlier flowering phenotype than the wild type under long-day conditions, whereas the *myc2*, *myc3*, and *myc4* single mutants and the *myc3/4* double mutant had similar flowering times to the wild type. MYC2 is also involved in the developmental regulation of the calyx (Qi et al., 2015a). The *myc2, myc3, myc4*, and *myc5* mutants displayed a failure to dehisce anther phenotype, delayed dehiscence, and low pollen grain survival, whereas the quadruple mutants had a delayed calyx development phenotype. Floral spike is an important component of the floral organ that affects reproductive growth and crop yield (Cai et al., 2014). EXTRA GLUME 1 and 2 (EG1 and EG2) were identified from spike deficient mutants of rice, where EG2 worked as a negative regulator to reduce MYC2-mediated floral morphogenesis. In the presence of JA signals, MYC2 promotes normal flowering spike by activating the flowering-related *MADS1*.

Ethylene is a regulatory molecule that promotes fruit ripening and organ senescence. In apples, JA induction and MYC2 can accelerate the expression of *1-AMINOCYCLOPROPANE-1-CARBOXYLATE OXIDASE* (*ACO*) and *1-AMINOCYCLOPROPANE-1-CARBOXYLATE SYNTHASE* (*ACS*), on the other hand, ethylene production (Li et al., 2017). MYC2 not only bond to the promoters of *ACS1* and *ACO1* to increase their expression levels, but also simultaneously increased the activity of ERF3, which mediated the transcription regulation of *ACS1* (Hu et al., 2022). MYC2 suppressed the repressive effect of ERF2 on the *ACS1* promoter by interacting with ERF2, which prevented ERF2 from binding to ERF3 and produced large quantities of free ERF3 to activate the transcription of *ACS1* (Kazan, 2015; Li et al., 2016). Interestingly, the IIIe bHLHs and the IIIb bHLH transcription factors bHLH3, bHLH13, bHLH14, and bHLH17 have antagonistic roles in regulating physiological processes such as chlorophyll accumulation, photochemical efficiency of PHOTOSYSTEM II (PSII), and membrane ion permeation (Qi et al., 2015b).

Leaf senescence is regulated by a large number of internal and environmental factors. MYC2, MYC3, and MYC4 are capable of activating JA-induced leaf senescence and regulating the senescence process by binding to the promoter of *SENESCENCE-ASSOCIATED GENE29* (*SAG29*), whereas IIIb bHLHs bind to the *SAG29* promoter to reduce its expression (Qi et al., 2015b). H_2_O_2_ scavenging significantly inhibits JA-induced leaf senescence and the expression of *SAGs*. Some studies have shown the *myc2* mutant exhibits delayed leaf senescence with increased expression of *CATALASE 2* (*CAT2*) and lower H_2_O_2_ accumulation (Zhang Y. et al., 2020). Arabidopsis DNA binding-with-one-finger (Dof) proteins are associated with leaf senescence. *Dof2.1*, a JA-inducible gene, accelerated leaf senescence by increasing the expression of *MYC2*. MYC2 was also responsible for the JA-inducible expression of *Dof2.1* (Zhuo et al., 2020). U-box Senescence Related 1 (USR1) is involved in both age-dependent and dark-induced leaf senescence in Arabidopsis. The *usr1* mutant exhibited a delay in the age-dependent and dark-induced leaf senescence. JA treatments promoted the accumulation of *AtUSR1* by the activation function of MYC2 (Zhang Z. et al., 2020).

## MYC2 Participates in the Biotic Stress Response

**FIGURE 4:**
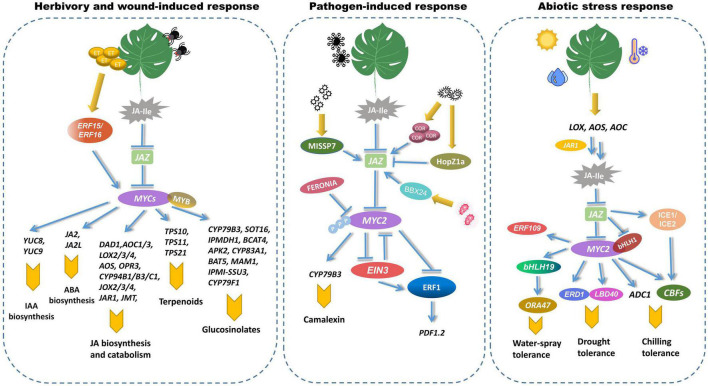
MYCs regulate biotic and abiotic stress responses in plants. MYCs participate in the process of pathogen infection and wound responses. MYCs interact with MYB to control the expression of several glucosinolate-biosynthetic genes, such as *CYP79B3*, *SOT16*, *IPMDH1*, and *BCAT4*, etc. (Mikkelsen et al., 2003; Piotrowski et al., 2004; Knill et al., 2008; He et al., 2011). MYC2 promotes the production of terpenoid phytolexin by upregulating the expression of *TPS10, TPS11*, and *TPS21* (Hong et al., 2012). MYC2 regulates the associated mechanical injury of herbivorous insects by positively regulating the expression of *NAC019* and *JA2L*, which increase the expression level of *VSP1* and *TD* (Du et al., 2014, 2017). MYC2 enhanced the transcription of the IAA biosynthesis genes *YUC8* and *YUC9* (Pérez-Alonso et al., 2021). The transcription of multiple JA biosynthesis and catabolism genes are also mediated by MYC2 under wounding response. MYC2 interacts with EIN3 in ethylene signaling to antagonistically regulate the expression of *ERF*, which in turn regulates the expression of pathogen-related *PDF1.2* (Song et al., 2014). MiSSP7 and BBX24 inhibit MYC2-targeted gene expression by increasing their interaction with JAZ (Daguerre et al., 2020; Zhang H. et al., 2020). HopZ1a triggered AtJAZ1 degradation and increased the expression of JA-responsive genes (Jiang et al., 2013). MYC2 function is inhibited by FER via its phosphorylation (Guo et al., 2018). MYCs are engaged in abiotic stresses such as low temperature, water-spray and drought. Environmental stress can increase the transcription of *MYC2* and *bHLH1*, which in turn upregulate the expression of low-temperature stress-related *CBFs* and drought stress-related *ERD1* (Li Y. et al., 2019; Wang Y. et al., 2019). ICE1 and ICE2 upregulate the expression of *CBFs* (Kashyap and Deswal, 2019). MYC2 increased *ADC1* levels, which resulted in increased putrescine biosynthesis (Ding et al., 2021). Water spray-inducible MYC-regulon creates a hierarchical transcriptional network via interacting with bHLH19 and ERF109 (van Moerkercke et al., 2019). bHLH19 in turn directly activates the promoter of *ORA47* (Chen et al., 2016).

MYC2, MYC3, and MYC4 play analogous roles in regulating plant resistance to insects (Figure 4). The *myc2 myc3 myc4* triple mutant is more susceptible to *Spodoptera mauritia* and *Spodoptera exigua* Hiibner than the wild plant (Fernández-Calvo et al., 2011; Schweizer et al., 2013; Song et al., 2014). MYC2 regulates the associated mechanical injury caused by herbivorous insects to positively regulate the expression of *JASMONIC ACID2* (*JA2*) and *JASMONIC ACID2-LIKE* (*JA2L*), therefore controlling ABA biosynthesis and stomatal closure (Du et al., 2014). MYC2 promotes the transcription of the IAA biosynthesis gene *YUCCA* induced by the bite of the two-spotted spider mite *Tetranychus urticae* by binding to the tandem G-box in the promoter of *YUC8* and *YUC9* (Pérez-Alonso et al., 2021). MYC2, MYC3, and MYC4 promote the content of glucosinolates to enhance insect resistance. Glucosinolates are a class of thioglucosides containing nitrogen and sulfur, whose biosynthesis were governed by six R2R3-MYB transcription factors: MYB28, MYB29, MYB76, MYB34, MYB51, and MYB122. The interaction of MYCs and MYBs promotes the transcription of glucosinolate-related genes, resulting in increased GS levels (Schweizer et al., 2013). By forming a transcription factor complex with several MYB transcription factors, MYC2, MYC3, and MYC4 bond to the promoters of more than twenty glucosinolate biosynthesis genes, and directly regulated their expressions (Schweizer et al., 2013). In addition, MYC2 binds directly to the promoters of the *terpene synthases TPS10*, *TPS11*, and *TPS21*, and triggers their transcription. This could boost terpenoid production and provide independent resistance to insect attacks (Hong et al., 2012). Wounding-induced JA biosynthesis was relevant to the transcriptional reprogramming on the downstream of JA biosynthesis. JA catabolic genes, such as *allene oxide synthase* (*AOS*), *allene oxide cyclase1 (AOC1), cis-(*+)*-12-oxo- phytodienoic acid reductase 3(OPR3), lipoxygenase 2 (LOX2), JASMONATE RESISTANT 1 (JAR1), jasmonic acid carboxyl methyltransferase (JMT), jasmonate-induced oxygenases 2 (JOX2)*, and *cytochrome P450 94B1 (CYP94B1)*, etc. are upregulated by MYC2 at different levels (Zhang C. et al., 2020). ET-induced ERF15 and ERF16 also regulate JA biosynthesis in tomato. ET triggered the transcription of MYC2 and ERF16 by regulating JA biosynthesis genes (Hu et al., 2021).

In contrast to its regulatory role in insect resistance, MYC2 serves as a negative regulator of plant pathogens (Zheng et al., 2017). MYC2 blocked the function of EIN3 by decreasing ethylene-mediated pathogen response. MYC2 interacts with EIN3 to antagonistically regulate the expression of *ERF*, which enhanced the expression of pathogen-induced *PDF1.2* (Song et al., 2014). The *myc2* mutants are more resistant to pathogens like *Botrytis cinerea* than the wild type, probably attributed to MYC2 reciprocally inhibiting the function of EIN3. Cytochrome P450 enzymes are required for camalexin biosynthesis. CYP79B2 or CYP79B3 convert Trp to indole-3-acetaldoxime. Pathogen infection or abiotic stresses could induce the expression of *CYP71A12* and *CYP71A13*, which dehydrate indole-3-acetaldoxime to produce indole-3-acetonitrile (IAN). IAN is subsequently activated, probably to indole cyanohydrin, and conjugated with glutathione to form GS-IAN. GS-IAN is degraded to Cys (IAN) via glutamyl peptidase 1 (GGP1), and Cys (IAN) is then converted to camalexin via *CYP71B15* (Koprivova et al., 2019; Mucha et al., 2019). MiSSP7, a fungal symbiotic effector from *Laccaria bicolor*, blocked the dimerization of Populus JAZs, which facilitated the release of MYC2-targeted genes (Plett et al., 2014; Daguerre et al., 2020). The *jin1* mutants are resistant to both *P. syringae* pv. tomato DC3000 and COR. The *JIN* gene is necessary for the inhibition of salicylic acid-dependent defenses in *P. syringae* infection. COR activates NAC transcription factors via MYC2, impairing the production of salicylic acid (SA) and weakening host immunity (Laurie-Berry et al., 2006). By phosphorylating and degrading MYC2, the receptor kinase FERONIA (FER) suppresses JA and COR signaling, hence positively regulating immunity (Guo et al., 2018). Transcriptional activation of the SA hormone defense sector acts as a critical barrier to biotrophic infections. In Arabidopsis effector-triggered immunity, the EDS1 complex in combination with PAD4 inhibits MYC2 to enhance the SA defense (Cui et al., 2018). *P. syringae* HopZ1a induced the degradation of AtJAZ1, and fostered JA-responsive gene expression during bacterial infection (Jiang et al., 2013). HopZ1a acetylated JAZs through a putative acetyltransferase catalysis. In recent studies, MYC2 has been implicated as a negative regulator of SA-mediated defense against bacterial infections. Overexpression of *MYC2* confers hyper-resistance to a wide range of virulent and avirulent bacterial pathogens (Gautam et al., 2021). IbBBX24, a transcription factor from the B-box (BBX) family, modulated JA responses in sweet potato by antagonistically binding to IbJAZ10, which interacted with IbMYC2 (Zhang H. et al., 2020). Overexpression of *IbBBX24* dramatically enhanced resistance to Fusarium wilt disease. Using the ChIP-seq approach, 665 MYC2-targeted binding genes in tomato were discovered (Du et al., 2017). These genes were found to be highly abundant under early JA stress, and one type of MYC2-targeted transcription factor (MTF) directly regulated the transcription of late defense genes.

## MYC2 Participates in the Abiotic Stress Response

Beyond modulating biotic stress responses, MYC2 is engaged in abiotic resistance, such as cold and drought stress (Figure 4). To avoid freezing injury or death, plants bolster the expression of *C-repeat-binding factor* (*CBF*), *cold-regulated (COR), cold-induced (KIN)*, and *low-temperature-induced (LTI)*. The INDUCER OF CBF EXPRESSION (ICE)-CBF transcriptional cascade is critical for the plant cold stress response (Kashyap and Deswal, 2019). ICE1 and ICE2, two bHLH TFs from *A. thaliana*, upregulate the expression of *CBF* by binding to CANNTG motif in the promoter. At the normal temperature, JAZ1/4 interacts with ICE1 and ICE2 to block the ICE-CBF pathway. However, Low-temperature conditions promote the expression of JA biosynthesis-related genes such as *AOS1*, *DAD1*, *AOC*, and *LOX2*, resulting in the production of JA-Ile and stimulation of COI1 to interact with JAZ1 (Zhu, 2016; Hu et al., 2017). Putrescine alleviated the oxidative damage caused by cold stress. Some research has demonstrated that inhibition of MYC2 decreased the expression of *arginine decarboxylase 1* (*ADC1*) and lowered putrescine accumulation (Ding et al., 2021). JA regulated chilling response in apple (Wang Y. et al., 2019). Exogenous application of MeJA could increase the expression of *bHLH1*, *CBF1*, *CBF2*, and *CBF3*. bHLH1 regulated the chilling resistance by interacting with JAZ1/4 and MYC2. MYC2 sharply increased the expression level of *CBFs*, whereas JAZ1/4 decreased the facilitation of MYC2 on chilling resistance. Furthermore, MYC2 participated in JA-mediated drought tolerance (Li Y. et al., 2019). In *A. thaliana*, the *coi1-2*, *JAZ1-Jas*, and *myc2-2* mutants exhibit drought sensitivity and regulate the stress caused by water deficit through the induction of *EARLY RESPONSIVE TO DEHYDRATION 1* (*ERD1*). MYC2 modulates drought-responsive genes by binding to the promoter of *ERD1*, which in turn enhances drought tolerance. JA and 12-OPDA could increase stomatal closure. The elevation in OPDA levels coordinated with the decreased stomatal aperture and the increased drought tolerance (Savchenko et al., 2014). OsbHLH148 binds to OsJAZ1 to induce the expression of *OsDREB1*, thereby enhancing the drought resistance in rice (Sarwat and Tuteja, 2017). Recent studies have demonstrated that the Arabidopsis BBD proteins are essential in the ABA-mediated interplay of ABA and JA against drought stresses. Overexpression of *AtBBD1* increased ABA sensitivity considerably and enhanced the expression of critical genes involved in the ABA-mediated drought cascade, particularly those related to ABA biosynthesis and downstream transcription factors (Mahmudul Huque et al., 2021). The LATERAL ORGAN BOUNDARIES DOMAIN (LBD)-containing protein isolated from tomato participates in JA-mediated drought tolerance (Liu et al., 2020). The knockout of *SlLBD40* improved the water-holding ability of tomatoes. Water spray-inducible MYC-regulon creates a hierarchical transcriptional network via interacting with bHLH19 and ERF109, which demonstrated that spray-induced jasmonate accumulation was transcriptionally regulated by an MYC2-controlled positive-feedback loop (van Moerkercke et al., 2019).

## MYC2 Regulates the Biosynthesis of Plant Secondary Metabolites

**FIGURE 5:**
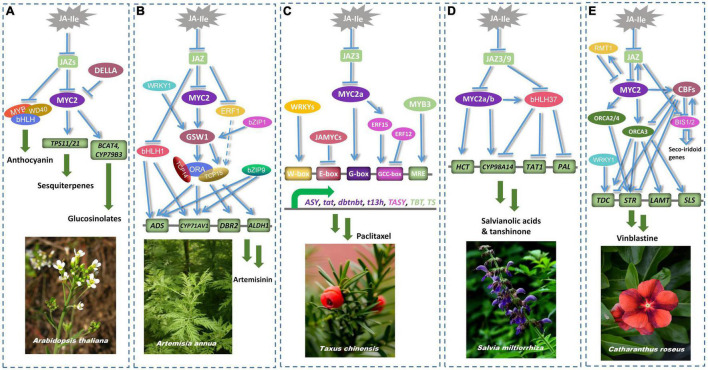
MYC2 plays an important regulatory role in the formation of secondary metabolites in model plants. **(A)** AtMYC2 alone or together with the MYB/bHLH/WD40 complex regulates the expression of *CYP79B3*, *TPS11*, *TPS21* and anthocyanins accumulation (Xu et al., 2013; Wang L. et al., 2019). **(B)** AaMYC2 up-regulates the expression of *CYP71AV1*, by regulating the transcription of the glandular trichome-specific transcription factor AaGSW1 (Yu et al., 2012). AaMYC2 cooperates with ERFs, bHLHL1 and WRKY1 to jointly regulate the expression of *ADS* and *DBR2* (Ma et al., 2018; Li L. et al., 2019). **(C)** TcMYC2a interacts with JAZ3 protein to regulate the expression of *tat*, *dbtnbt*, *t13h*, and coordinates with ERF12/15 to control the expression of *TASY* (Zhang M. et al., 2015; Zhang et al., 2018b). TcMYC2 and TcJAMYCs antagonistically regulate key genes of paclitaxel biosynthetic pathway (Lenka et al., 2015). **(D)** SmMYC2 interacts with SmJAZ3/9 to competitively control the expression of *HCT6* and *CYP98A14* (Zhou et al., 2016). bHLHL37 and MYC2 synergistically regulate the synthesis of salvianolic acid B. Unlike MYC2, bHLHL37 negatively regulates the expression of *TAT1*, *PAL1*, and *CYP98A14* (Du et al., 2018). **(E)** CrMYC2 affects the expression of downstream genes *STR* and *TDC* by regulating the transcription of *ORCA2* and *ORCA3* (Van der Fits and Memelink, 2001; Zhang et al., 2011). In contrast, the negative regulators CBF1 and CBF2 competitively bind to MYC2-targeted genes thereby repressing the expression of TIA pathway genes (Sui et al., 2018).

Plants have evolved over time to develop a variety of stress-responsive mechanisms, including the production of defense chemicals against external stimuli. When a plant is stressed or induced by hormones, numerous secondary metabolites arise and accumulate. JA regulates the expression of key enzymes involved in plant secondary metabolism by activating or suppressing the activity of relevant transcription factors, which influences the expression of key enzymes and, as a result, influences secondary metabolite accumulation (Chen et al., 2019). In general, different types of transcription factors specifically regulate the biosynthetic pathways of different secondary metabolites, and some transcription factors are usually capable of regulating multiple genes along the same route (Figure 5).

MYC-like transcription factors are involved in the regulation of secondary metabolites such as terpenoids, alkaloids, flavonoids, and thioglycosides (Afrin et al., 2015). Paclitaxel, artemisinin, and tanshinone are terpenoids, which are classified into monoterpenes, sesquiterpenes, diterpenes, and triterpenes based on the number of isoprenoid units. AtTPS21 and AtTPS11 are responsible for volatile sesquiterpene biosynthesis. AtMYC2 directly binds to the promoters of *TPS11* and *TPS21* to regulate their expressions (Hong et al., 2012). DELLA can interact with MYC2 to repress the transactivation of *TPS*. Artemisinin is a sesquiterpene lactone whose biosynthesis is restricted to the specific glandular trichome cells (Olsson et al., 2009). Artemisinin biosynthesis genes are regulated by octadecanoid-derivative responsive AP2-domain protein (AaORA), which is regulated by the glandular trichome-specific transcription factor *Artemisia annua* GST-specific WRKY1 (AaGSW1). Overexpression of *AaMYC2* resulted in large increases in *cytochrome P450–dependent hydroxylase (CYP71AV1)* and *double bond reductase 2* (*DBR2*) levels, as well as an elevation in artemisinin content (Yu et al., 2012). MYC2 and bZIP1 synergistically regulate the expression of *GSW1*, while GSW1 binds to the promoter of *TEOSINTE BRANCHED 1/CYCLOIDEA/PROLIFERATING CELL FACTOR15* (*TCP15*) to regulate its transcription. ORA interacts with TCP14/15 to enhance the expression and transactivation of *DBR2* and *ALDEHYDE DEHYDROGENASE 1* (*ALDH1*) via TCP14/15 (Ma et al., 2018, 2021). AabHLH1 was specifically expressed in glandular trichome, and overexpression of bHLH1 can increase the expression of *AMORPHA-4, 11-DIENE SYNTHASE* (*ADS*), and *CYP71AV1* (Li L. et al., 2019). AabZIP9 binds to the ACGT element on the promoters of *ADS* and *CYP71AV1* (Shen et al., 2019). Overexpression of AabZIP9 increased the expression of *ADS*, but had no significant effect on the expression of *CYP71AV1*, *DBR2*, and *ALDH1*.

Paclitaxel is a diterpene that is strongly triggered by JA. Extensive *in vitro* interaction studies revealed that MYCs were involved in the expression of several critical genes in the upstream of paclitaxel (Cui et al., 2019). TcMYC2a can interact with JAZ3 protein to mediate the expression of paclitaxel biosynthesis genes. By binding to T/G-box, G-box, and E-box, TcMYC2a affects the expression level of the *GUS* gene. Overexpression of *MYC2a* in the *Taxus chinensis* suspension cell line increases the expression of *taxadiene synthase* (*TASY*), *taxadiene-5*α*-ol-acetyltransferase* (*tat*), *30-N-dephenylated paclitaxel-N-benzoyltransferase* (*dbtnbt*), and *taxane 13-alpha-hydroxylase* (*t13h*), whereas TcMYC2a inhibits the expression of *TASY* through its interaction with TcERF12/15 (Zhang M. et al., 2015; Zhang et al., 2018b). JA-inducible MYC transcription factors (TcJAMYC1, TcJAMYC2, and TcJAMYC4) are a group of negative regulators induced by JA whose domains are highly homologous to AtMYC2 (Lenka et al., 2015). JAMYCs regulate paclitaxel biosynthesis via binding to the E-box on the promoters of paclitaxel-biosynthetic genes *TASY, taxadien-5*α*-ol by taxadien-5*α*-ol hydroxylase(T5aH), 10-deacetyl baccatinIII-10-O- acetyltransferase(DBAT), taxane*α*-O-benzoyltransferase(DBBT), phenylalanine aminomutase (PAM), Baccatin III:3-amino, 3-phenylpropanoyl transferase(BAPT), 3-N-debenzoyl-2-deoxypaclitaxel-N-benzoyltransferase(DBTNBT)*. Overexpression of JAMYC negatively regulates the transcription of key genes related to paclitaxel biosynthesis. TcWRKYs regulate the expression of *TcERF12/15* and paclitaxel-biosynthetic genes at different levels (Zhang et al., 2018a). Overexpression of *WRKY8/20/26/47* positively regulates the expression of paclitaxel-biosynthetic genes. TmMYB3 increases the production of the paclitaxel precursor 10-deacetylbaccatin by binding to the MRE elements to upregulate the expression of *TBT* and *TS* (Yu et al., 2020).

Tanshinone is a representative diterpenoid. Tanshinone and salvianolic acid are two important active components in *Salvia miltiorrhiza*, whose biosynthesis is induced by JA. *SmMYC2a* and *SmMYC2b* participate in JA-mediated biosynthesis of tanshinone and salvianolic acid (Zhou et al., 2016). Hydroxy-cinnamoyl transferase6 (SmHCT6) and cytochrome P450 monooxygenase (SmCYP98A14) are key enzymes of the salvianolic acid biosynthesis pathway, whose expressions are induced by JA. MYC2a activates the expression of *HCT6* and *CYP98A14* by attaching to the promoter E-box of *HCT6* and *CYP98A14*. MYC2b regulates the expression of *CYP98A14* by binding to the E-box of *CYP98A14*. On the contrary, overexpression of *SmJAZ3* or *SmJAZ9* in *S. miltiorrhiza* hairy roots resulted in a significant decrease in tanshinone content, which might be attributed to the interaction of JAZ proteins with MYC2 to inhibit their transactivation. Tyrosine aminotransferase1(*TAT1*), phenylalanine ammonialyase1(*PAL1*) and *CYP98A14* are key genes for tanshinolate biosynthesis. *SmbHLHL37* and *SmMYC2* synergistically regulate the synthesis of salvianolic acid B (Du et al., 2018). The expression of *bHLHL37* is regulated by MYC2. It was found that MYC2 regulates the transcriptional activity of salvianolic acid B biosynthesis genes by binding to their promoters. bHLHL37 can dimerize and interact with JAZ3/8. Overexpression of *bHLHL37* decreases the synthesis of salvianolic acid B. bHLHL37 inhibits the production of salvianolic acid B by binding to the promoters of *SmTAT1*, *SmPAL1*, and *SmCYP98A14* to suppress their transcriptional activities.

Terpene alkaloids, especially terpene indole alkaloids (TIAs), have received more attention in *Catharanthus roseus*. JA is primarily responsible for inducing the production of these alkaloids. CrMYC1 was altered by fungal elicitor and JA in *C. roseus* suspension cells, indicating that CrMYC1 may be involved in the regulation of genes involved in JA signaling (Chatel et al., 2003). CrMYC2 is an early JA-responsive factor and regulates the expression of a series of TIA biosynthetic genes, by modulating the transcription of *octadecanoid-derivative responsive Catharanthus AP2-domain proteins* (*ORCA2* and *ORCA3*) (Zhang et al., 2011). Knockdown of *CrMYC2* by RNA interference significantly reduced the expression of *ORCA2* and *ORCA3.* MeJA induced the expression of *ORCA2* and *ORCA3* and was specifically bound to the JERE element in the *strictosidine synthase* (*STR*) promoter. This implies that CrMYC2 stimulates STR activity via binding to a G-box-like element on the promoter of *ORCA2/3*, triggering the production of ORCAs-targeted genes rather than directly to the *STR* promoter. CrMYC2 also controls the expression of TIA biosynthesis genes in collaboration with certain repressors. The G-box binding factors CrCBF1 and CrCBF2 act as negative regulators to repress the production of TIAs (Sui et al., 2018). Overexpression of *CrMYC2* increased the expression of *CrGBF* and eventually led to a reduction in alkaloid accumulation. CrGBF1 and CrGBF2 can form homo- and heterodimers to repress the activation of TIA pathway genes. CrGBF dimerizes with CrMYC2 and suppresses the transactivation of alkaloid-bisynthetic genes by competitively binding to the T/G-box of *CrMYC2* targeted genes. Like CrGBF, *CrRMT1* acts as a repressor of CrMYC2 that negatively regulates the MYC2-mediated transactivation of TIA-related genes (Patra et al., 2018). Nicotine is another well-studied alkaloid, featuring *NtCOI1*, *NtJAZ*, and *NtMYC2* involved in its production. Putrescine N-methyltransferase (NtPMT1a) is a key enzyme in nicotine production. NtMYC2 controls nicotine production via two regulatory mechanisms (Shoji and Hashimoto, 2011). The first directly binds to the G-box of the *PMT1a* promoter to regulate its transcriptional activation, and the second regulates the expression of *PMT1a* through interacting with ERF in the NIC2 region. NtMYC2a and NtMYC2b form a conjugated complex with NtJAZ1 that negatively regulates multiple steps of JA-induced nicotine biosynthesis (Zhang et al., 2012). Our research investigated the TIA-regulatory gene *MYC2* in *Dendrobium officinale* (Zhu et al., 2017). *DoMYC2* negatively regulates the upstream pathway of terpene alkaloids. The expression of *DoMYC2* could be induced by MeJA and chitosan in a short period. Overexpression of *DoMYC2* in Arabidopsis attenuated the expression levels of *HMG-CoA reductase* (*AtHMGR2*), *farnesyl diphosphate synthase* (*AtFPS1/AtFPS2*), and *isopentenyl transferases* (*AtIPTs*).

## Conclusion and Future Challenges

Since the discovery of JA receptor COl1, several regulatory factors in the JA signaling pathway have been uncovered one after another. JA plays an essential role in both maintaining normal development and protecting plants from environmental stresses. Plants have evolved a sophisticated negative feedback mechanisms to regulate the moderate onset of resistance responses during their continuous adaptation to the environment and various stresses. In this review, we encapsulate the diverse roles of MYC TFs and generalize a MYC-mediated JA signaling pathway. This gives our understanding of how JA works to maintain a balance between plant growth and adaptability to the external environment. The basic guidelines described here provide a framework for elucidating a cross-regulation between MYC2 and its co-regulons. As the core regulatory molecules of the JA signaling branch, MYC2 and its relatives directly target hundreds of JA-responsive genes, building a positive cascade of transcriptional reprogramming. The downstream TFs under MYC2 and its homologs directly targeted overlapping but separate cohorts of genes, indicating that they have different roles within the JA response. The intricacy within the MYC2-mediated JA signaling pathway in general is the presence of some feedforward and feedback loops that are simultaneously activated. However, relationships between these subnetworks through the dynamics and resilience of their regulatory effects on the broader network are not well understood. Uncoupling these subnetworks would be an effective way to assess how they socialize to drive a robust activation of JA signaling.

## Author Contributions

CS, YC, and HD discussed the writing plan. CS, YC, JD, and GL drafted the manuscript. CS, MM, and CC edited the manuscript. HD and CS acquired the funding. All authors have read, reviewed, and approved the submitted version.

## Conflict of Interest

The authors declare that the research was conducted in the absence of any commercial or financial relationships that could be construed as a potential conflict of interest.

## Publisher’s Note

All claims expressed in this article are solely those of the authors and do not necessarily represent those of their affiliated organizations, or those of the publisher, the editors and the reviewers. Any product that may be evaluated in this article, or claim that may be made by its manufacturer, is not guaranteed or endorsed by the publisher.
